# Supramaximal interval training using anaerobic speed reserve or sprint interval training in rowers

**DOI:** 10.3389/fphys.2025.1516268

**Published:** 2025-03-27

**Authors:** Yu Tongwu, Zhong Jinghui, Ding Chuanwei, Zhang Zijian, Xu Yuxiong

**Affiliations:** ^1^ Anhui Communications Vocational and Technical College, Hefei, China; ^2^ Capital University of Physical Education and Sports, Beijing, China

**Keywords:** high-intensity interval training, sprint interval training, aerobic fitness, anaerobic power, performance variability, rowing

## Abstract

**Objective:**

This study aimed to compare the consistency of physiological adaptations and inter-individual variability in response to three distinct high-intensity interval training (HIIT) protocols—anaerobic power reserve (APR), maximal aerobic power (MAP), and sprint interval training (SIT)—among elite male rowers. By exploring the impact of individualized intensity prescriptions, we sought to identify the most effective protocol for enhancing training consistency, as well as improving both aerobic and anaerobic performance while minimizing variability in individual responses.

**Methods:**

Thirty well-trained male rowers (mean age: 24.9 ± 3.1 years; height: 185 ± 4.4 cm; body mass: 86 ± 7.9 kg; body fat: 12.5% ± 2.4%) participated in the study. All participants were members of a national rowing team with an average of 6 years of competitive experience and regular participation in national and international championships. The intervention involved 6 weeks of individualized HIIT, performed three times per week, with pre- and post-tests assessing VO_2_max, cardiovascular efficiency (Qmax), anaerobic power (MSP, CP), and 2,000-m rowing performance.

**Results:**

All interventions resulted in significant improvements in VO_2_max, Qmax, MSP, and 2,000-m rowing time trial performance (p < 0.05). The SIT group exhibited the largest relative improvements, with VO_2_max increasing by 6.3% (from 51.9 ± 3.2 to 55.2 ± 3.3 mL·kg^-1^·min^-1^, Cohen’s d = 1.05, 95% CI [0.57, 1.53]), Qmax by 6.4% (Cohen’s d = 1.15, 95% CI [0.66, 1.64]), and a 3.7% reduction in 2,000-m time (Cohen’s d = 0.86, 95% CI [0.39, 1.33]). Notably, SIT demonstrated the lowest variability across all measured outcomes, as evidenced by reduced coefficients of variation and narrower confidence intervals.

**Conclusion:**

The SIT protocol, emphasizing maximal exertion, led to the most consistent adaptations and the greatest improvements across key performance metrics, including VO_2_max, Qmax, and 2,000-m rowing performance. These results suggest that SIT may be the optimal approach for improving performance consistency and maximizing physiological adaptations in elite rowers. Future research should explore the long-term applicability and potential integration of SIT with other training modalities to further enhance rowing performance.

## Introduction

High-intensity interval training (HIIT) is widely regarded as an effective method for enhancing athletic performance, with numerous studies demonstrating its significant effects on improving cardiorespiratory function, muscle strength, and metabolic health (Faelli et al., 2022). However, individualized training programs still face challenges in reducing variation in adaptation responses (Mann et al., 2014). Given the different physiological characteristics and training backgrounds of athletes, ensuring standardized training intensity while effectively accommodating individual differences remains an unresolved issue (Mann et al., 2014; Bossi et al., 2023; Bassett and Howley, 2000).

In recent years, to address these challenges, research has increasingly focused on supramaximal sprint interval training (SIT). SIT is a high-intensity training method performed over a short duration, aimed at maximizing anaerobic capacity and neuromuscular efficiency. It has been shown to significantly improve fitness in rowers and other competitive athletes (Thurlow et al., 2024). For example, Jung et al.'s study found that SIT with short rest periods could significantly enhance initial explosive acceleration while maintaining sprint mechanics, which is crucial for improving athletes’ explosive power and repeated sprint ability (Jung and Hong, 2024). Sprint capacity is closely related to anaerobic metabolic efficiency and the recruitment of fast-twitch muscle fibers, which are essential for providing high-intensity energy during short durations (Myrvang and van den Tillaar, 2024).

Repeated sprint training (RST), another common high-intensity training method, has also demonstrated significant effects on enhancing athletes’ physical fitness and physiological adaptations (Di Prampero, 1985). Studies indicate that adjusting RST parameters (such as frequency, sprint distance, and repetitions) can significantly improve aerobic capacity, sprint speed, repeated sprint ability (RSA), and agility (Holt et al., 2019; Sandford et al., 2021). Systematic reviews and meta-analyses further support these findings. Thurlow et al.'s study highlighted that RST can significantly increase VO_2_max, sprint times, and RSA. This high-intensity training model not only effectively improves cardiorespiratory function but also enhances neuromuscular responsiveness, thereby improving overall athletic performance (Thurlow et al., 2024).

Additionally, resisted sprint training has been shown to significantly enhance athletes’ acceleration ability. Compared to traditional sprint training, resisted sprints increase ground resistance, prompting athletes to generate greater horizontal ground reaction forces during the sprint, thereby improving short-distance acceleration. The combination of resistance and assisted sprints can significantly improve maximum velocity, suggesting that a combination of different sprint training types can effectively enhance athletes’ performance at various stages (Myrvang and van den Tillaar, 2024).

Moreover, the relationship between vertical jump ability and sprint performance has also garnered significant attention. Washif et al.'s research showed a significant correlation between vertical jump height and sprint distance, particularly during the maximum velocity and takeoff phases (Washif and Kok, 2022). This finding emphasizes the necessity of combining strength training with speed training, especially for short-distance sprints that require high explosive power and quick response capabilities (Washif and Kok, 2022). Additionally, a systematic review by Myrvang and van den Tillaar indicated that the combination of resisted and assisted sprint training had significant effects on improving different phases of sprint performance, further validating the importance of integrating multiple training methods (Myrvang and van den Tillaar, 2024).

Based on the above discussion, this study aims to explore the impact of individualized HIIT on the physiological adaptation of rowers, particularly the potential of SIT to reduce variability in training outcomes. We hypothesize that, due to the high-intensity demands of SIT, it will lead to more consistent adaptive responses and significantly improve anaerobic capacity and cardiorespiratory adaptations, thereby providing a scientific basis and practical guidance for individualized training (Mann et al., 2014; Bossi et al., 2023; Meyler et al., 2023; Bossi et al., 2023; Bagger et al., 2023; Thurlow et al., 2024).

## Methods

### Study design

The study was designed as a randomized controlled trial to assess the effects of 6 weeks of HIIT on thirty well-trained male rowers, utilizing three different individualized training protocols: G-APR, G-MAP, and G-SIT. The aim was to determine the effectiveness of each protocol in enhancing key physiological and performance metrics relevant to competitive rowing. Participants were tested at baseline and after the intervention for changes in VO_2_max, anaerobic performance (sprint power and lactate thresholds), and 2,000-m rowing performance, which is a critical indicator for rowers. The intervention was designed to provide insight into the specific adaptations elicited by different HIIT modalities, focusing on tailoring the intensity and type of exercise to each rower’s capabilities in order to optimize performance and reduce variability in training responses. This design allowed for a detailed evaluation of how targeted HIIT prescriptions could enhance both performance and physiological adaptations in elite athletes (Laursen and Buchheit, 2019a; Sandford et al., 2021; Buchheit and Laursen, 2013; Blondel et al., 2001).

### Participants

The study involved thirty well-trained male rowers (mean age: 24.9 ± 3.1 years; height: 185 ± 4.4 cm; body mass: 86 ± 7.9 kg; body fat percentage: 12.5% ± 2.4%), all of whom were members of a national-level team. These athletes had an average of 6 years of competitive rowing experience and regularly participated in high-level competitions, including national championships and international events. Participants were classified as elite according to the criteria outlined by McKay et al. (2021), which include: a minimum of 5 years of competitive training at the national level, consistent participation in international competitions, a high weekly training volume of 15–20 h, and a combination of strength, aerobic conditioning, and sport-specific skill training. This classification ensured that the findings could be generalized to similar elite athlete populations. Participants were selected to ensure a consistent training background, having undergone similar types of training regimens in terms of volume, intensity, and frequency. All participants provided written informed consent and passed a thorough medical screening (Laursen and Buchheit, 2019a; Sandford et al., 2021; Sheykhlouvand et al., 2022; de Almeida-Neto et al., 2022; Song and Sheykhlouvand, 2024).

### Testing sessions and location

All testing sessions were conducted in a temperature-controlled indoor facility at the Capital University of Physical Education and Sports Laboratory to maintain consistency. The environmental conditions were strictly controlled, with a temperature range of 20°C–22°C and relative humidity set between 45% and 55%, providing an optimal environment for assessing physical performance. The testing was scheduled in the morning (between 8:00 and 11:00 a.m.) to minimize the effects of circadian rhythms on physiological responses, as core temperature, hormone levels, and cardiovascular functions can fluctuate throughout the day (Hahn et al., 2000; Holt et al., 2019, Sandford et al., 2021).

The following tests were performed to assess the participants’ physical performance. The Rowing Ergometer Test involved participants performing a standardized rowing test on the Concept2 RowErg® rowing ergometer. During the test, participants rowed at a set intensity for a specific duration. The intensity was predetermined based on the individual’s MAP or APR to ensure that it was challenging but manageable. This test assessed endurance, peak power output, and overall rowing performance, as well as the participant’s ability to maintain consistent power output throughout the session.

The Lactate Threshold Test was conducted by measuring blood lactate concentrations during incremental exercise using the Lactate Scout + analyzer (EKF Diagnostics, Germany). The test followed a progressive exercise protocol, starting with low-intensity rowing that gradually increased in intensity. Blood samples were taken at regular intervals from the earlobe or fingertip to measure lactate accumulation. This test provided insights into anaerobic metabolism, identifying the point at which lactate accumulation exceeded the body’s ability to clear it, known as the lactate threshold. This is a key indicator of endurance performance and anaerobic capacity.

A Cardiorespiratory Test was performed using the MetaLyzer 3B-R2 system (Cortex Biophysik, Germany) to continuously monitor oxygen uptake (VO_2_) and ventilation. The exercise intensity was progressively increased to assess aerobic capacity and metabolic efficiency. Participants started at a low intensity, which was gradually increased every 2–3 min until exhaustion. Oxygen uptake and ventilation were measured continuously throughout the test, providing data on VO_2_max and overall cardiovascular fitness.

Cardiac Function Testing was performed using the PhysioFlow® transthoracic impedance cardiography system (Manatec Biomedical, France). This system continuously measured cardiac output, stroke volume, and heart rate during graded exercise. The exercise intensity was progressively increased in stages, allowing for the evaluation of cardiac function at various levels of exertion, from low-intensity warm-up to maximal effort. The PhysioFlow® system was calibrated before each session according to the manufacturer’s guidelines to ensure the accuracy of measurements.

By employing these standardized testing protocols and high-quality equipment, the study ensured accurate, reproducible, and reliable data collection. The combination of these tests allowed for a comprehensive assessment of both anaerobic and aerobic performance, as well as cardiovascular function during exercise, providing a holistic view of the participants’ fitness levels and adaptation to training.

### Rationale for prone position testing

Prone position testing was selected to isolate specific muscle groups relevant to rowing performance, particularly the lower back, gluteal muscles, and hamstrings, which are crucial during rowing strokes. The prone position allows for an optimal assessment of muscle function, especially in the posterior chain, which plays a significant role in rowing power and stability. This position minimizes compensatory movements from other muscle groups, such as the quadriceps and hip flexors, ensuring more accurate and reliable measurements of the key muscles involved in rowing. Additionally, rowers typically require substantial core and lower limb strength to maintain stability during powerful strokes; the prone position effectively targets these muscle groups, reflecting the demands of rowing. By using this approach, testing conditions are standardized, reducing variability in results and enhancing the reliability of the data collected (Coates et al., 2023).

### Ultrasound methodology

Muscle architecture was assessed using B-mode ultrasound (model LOGIQ e, GE Healthcare) with a linear-array transducer (frequency range: 10 MHz, resolution of 0.1 mm). Measurements were taken from the vastus lateralis muscle at 50% of femur length, following the European guidelines for muscle ultrasound assessment (Hahn et al., 2000). Participants were positioned in the prone position to ensure standardization. Three images were taken at each measurement site, and the average value of these images was used for analysis. All ultrasound measurements were performed by the same trained technician to ensure consistency and minimize inter-observer variability.

The methods for calculating the specific parameters were as follows: muscle thickness (MT) was measured as the distance between the outermost edges of the subcutaneous tissue and the deep aponeurosis of the muscle, taken perpendicular to the muscle fibers at the level of the muscle belly (Hahn et al., 2000). The pennation angle (PA) was determined by measuring the angle between the fascicles and the deep aponeurosis using image analysis software. For fascicle length (FL), the length of the muscle fibers was traced from their attachment points to the aponeurosis, with measurements taken using the linear scale provided by the ultrasound system. All measurements were repeated three times, and the average values were used for analysis to enhance measurement reliability and reduce potential errors (Laursen P. B. and Buchheit M., 2019).

### Reliability of key measurements

The reliability of key measurements in this study was ensured through the use of well-established testing procedures and equipment. To assess the consistency of our measurements, intra-class correlation coefficients (ICCs) for key variables were calculated. For VO_2_max, sprint power, and ultrasound measures (including muscle thickness, pennation angle, and fascicle length), ICC values were all above 0.90, indicating excellent reliability and minimal measurement error. This high ICC reflects that the data obtained from the testing sessions were consistent across repeated measurements, demonstrating the reliability of our instruments and testing protocols.

For key performance outcomes, such as rowing performance (2,000-m time trial), maximal sprint power (MSP), and anaerobic power, test-retest reliability was also evaluated (Vollaard et al., 2009; Bouchard et al., 2011; Coates et al., 2023). The coefficient of variation (CV) for these measures was consistently below 5%, suggesting that variation in repeated measurements was minimal, and that the observed changes in performance metrics were reliable indicators of physiological adaptations rather than inconsistencies in measurement.

To ensure the accuracy of the measurements, all testing procedures were s tandardized, and the same experienced technicians conducted the tests across all participants. The equipment, including the Concept2 RowErg® rowing ergometer, Lactate Scout + analyzer, and MetaLyzer 3B-R2 system, was calibrated prior to each session following the manufacturers’ guidelines. These careful calibration and procedural standardization minimized the potential for errors, allowing us to confidently attribute observed changes in performance and physiological responses to the training interventions rather than to measurement variability.

In addition to these statistical measures, the intervention was carefully designed to minimize any biases that could affect the reliability of the data. For example, power output during training was monitored in real-time via the rowing ergometer, and heart rate telemetry was used to ensure participants maintained the prescribed intensity during their training sessions (Sandford et al., 2021; Rosenblat et al., 2020; Wang and Zhao, 2023). These data were used in conjunction with other physiological responses to verify that the intensity levels were appropriately individualized for each participant.

### Statistical power calculation

A comprehensive *a priori* statistical power analysis was conducted using G*Power software (version 3.1) to determine the appropriate sample size for detecting significant changes in key physiological outcomes. The analysis was based on an estimated effect size of 0.40, derived from similar intervention studies involving elite athletes, with a power level (1-β) set at 0.80 and an alpha level (α) of 0.05. These parameters were selected to ensure a high likelihood of detecting true effects while minimizing the risk of Type I and Type II errors. The power analysis indicated that a minimum of 27 participants was required to achieve the desired statistical power (Saltin and Calbet, 2006; Levine, 2008; Iannetta et al., 2020). By recruiting 30 rowers, we provided a buffer to account for potential dropouts or unforeseen variability, thereby ensuring that the study maintained sufficient power to detect meaningful changes across the intervention groups. This careful planning was crucial, given the variability in adaptive responses often observed in training studies, particularly with elite athletes, where individual differences in trainability can influence outcomes. Therefore, a sample size of 30 was deemed both appropriate and robust for the purposes of this study, ensuring the reliability and validity of the results.

### Intervention protocols

Approximately 2 days after pre-intervention testing, participants began the first of their 6-week HIIT programs, completing three training sessions per week. Each training session was designed as follows: the G-APR group performed HIIT based on their anaerobic power reserve, completing two sets of 1-min rowing intervals, with the number of repetitions per set progressively increasing from 6 to 10 over the 6-week period, and intensity set at Δ30% APR. The G-MAP group rowed at 130% of their maximal aerobic power (MAP) in a similar two-set format with 1-min intervals. The G-SIT group completed a volume-matched protocol with maximum effort for each interval, rowing the equivalent distance to their Δ30% APR within 60 s.

### Exercise intensity monitoring and recovery duration

During each session, exercise intensity was monitored using heart rate telemetry (Polar H10) to ensure participants reached the required effort level. Additionally, power output was tracked via the Concept2 RowErg® to verify the consistency of supramaximal intensities. The recovery duration between efforts was set at 1 min, with a 3-min rest between sets to allow for adequate recovery without compromising subsequent performance.

### Training integration and seasonal context

The 6-week intervention replaced participants’ usual on-water rowing training but retained their off-water strength training to prevent detraining effects in these areas. This approach ensured that HIIT was the primary focus of their endurance training while maintaining their regular strength routines. The training program was conducted during the general preparation phase of the rowing season, specifically in the preseason, allowing participants to focus on physical conditioning without interference from competition schedules.

### Reproducibility and Transparency Considerations

To ensure reproducibility, all testing and training protocols were documented meticulously, with specific details on equipment settings, warm-up protocols, and timing of sessions. Each rower used the same ergometer for both pre- and post-tests to minimize inter-equipment variability. Personnel administering the tests were consistent throughout the study to further reduce variability.

### Cardiac function

Cardiac function was continuously monitored using non-invasive transthoracic impedance cardiography (PhysioFlow®, Manatec, France). Maximal cardiac output (Q̇max) and stroke volume (SVmax) were recorded continuously throughout the test. According to the manufacturer’s instructions, two electrodes were placed on the neck, two on the sternum, and one on each side of the chest. After a 20-s calibration, hemodynamic data were collected (Charloux et al., 2000).

### Determining anaerobic power and maximal sprint power

A 3-min all-out test was used to assess maximal sprint power (MSP), 60-s power (P60s), and critical power (CP). Following a self-paced warm-up, participants performed a 3-min row at 50 W, then immediately began the all-out test (Richer et al., 2016). The average power output was recorded from the start to the 10th second (MSP), from the start to the 60th second (P60s), and from the 150th to the 180th second (CP). The anaerobic power reserve was calculated as the difference between MAP and MSP.

### 2,000-m time trial performance

The 2,000-m rowing time trial was conducted on the same Concept2 rowing ergometer. The drag factor was individualized according to the manufacturer’s guidelines, adjusted to each participant’s weight category. After a self-paced warm-up and dynamic exercises, rowers completed the 2,000-m distance at maximum effort, with performance time recorded.

## Training programs

### Training protocol structure

The HIIT sessions were structured to maximize both safety and efficacy. Real-time power output measurements on the rowing ergometer were used to monitor external workload, ensuring participants consistently met prescribed intensity thresholds. Speed and cadence data were also tracked as additional metrics to confirm consistency in effort and rowing technique. Each HIIT session began with a standardized warm-up phase to prepare participants physically and mentally for the high-intensity efforts.

Warm-up Protocol: The warm-up consisted of 10 min of dynamic stretching, targeting major muscle groups involved in rowing, followed by low-intensity rowing to gradually increase muscle temperature, joint mobility, and heart rate. This phase was crucial for reducing injury risk and optimizing performance readiness. Dynamic stretching exercises focused on the shoulders, back, and lower body to ensure full range of motion and neuromuscular activation, essential for effective rowing performance (Thiele et al., 2020). The low-intensity rowing portion was performed at approximately 50% of each participant’s MAP to promote cardiovascular and muscular readiness without inducing significant fatigue.

Placement and Progression: High-intensity intervals were performed immediately after the warm-up to capitalize on the increased muscle temperature and readiness induced by the warm-up. Participants completed between 4 and 6 sets of 1-min intervals per session, with the number of sets increasing over the 6-week intervention to progressively overload the participants and stimulate further adaptation. Recovery periods of 1 min between intervals allowed for partial metabolic recovery while maintaining an elevated heart rate to promote cardiovascular adaptations (Thiele et al., 2020). In addition, a 3-min rest period between sets was incorporated to ensure participants could maintain high-quality efforts in subsequent intervals. The progression from 4 to 6 sets over the intervention was designed to gradually increase training load, promoting continued physiological adaptation while minimizing the risk of overtraining.

Sets, Repetitions, and Load Progression: Initial sessions involved four repetitions of 1-min intervals, which progressively increased to six repetitions by the later stages of the intervention. Each interval was performed at a prescribed power output corresponding to a specific percentage of APR, starting at 80% APR in weeks 1-2, increasing to 85% APR in weeks 3-4, and reaching 90% APR in weeks 5–6. This progressive increase in intensity and volume provided an effective training stimulus, challenging both anaerobic and aerobic energy systems while minimizing the risk of overtraining. The combination of increasing repetitions and higher intensity ensured a gradual and manageable progression, essential for optimizing adaptation and minimizing injury risk. Throughout the training, participants received real-time feedback on their power output and heart rate to help them maintain prescribed effort levels, ensuring consistency and maximizing the efficacy of the intervention.

Reproducibility and Transparency Considerations: To ensure the reproducibility of the study, detailed records were kept of all training and testing procedures, including warm-up components, interval durations, recovery periods, and intensity levels. The rowing ergometer settings were standardized across participants, and calibration was performed before each training session to minimize variability. Additionally, all testing was conducted under consistent environmental conditions, with temperature and humidity controlled. A summary table (Table 1) has been provided to illustrate the structure of the HIIT program, including sets, repetitions, intensity, and load progression. This table provides a clear overview of the intervention, ensuring transparency and enabling future researchers to replicate or build upon this study with confidence.

**TABLE 1:** Overview of training program structure.

Variable name	Week 1–2	Week 3–4	Week 5–6
Warm-up	Dynamic stretching + low-intensity rowing	Dynamic stretching + low-intensity rowing	Dynamic stretching + low-intensity rowing
Sets	4	5	6
Interval Duration	1 min	1 min	1 min
Recovery Period	1 min	1 min	1 min
Intensity (% APR)	80%	85%	90%

### Intensity monitoring and verification

To ensure accurate monitoring of training intensity during SIT, two primary methods were used: real-time power output tracking and heart rate monitoring. Power output was continuously recorded using the Concept2 RowErg® rowing ergometer, a validated tool for assessing rowing performance. Prior to each testing and training session, the rowing ergometer was calibrated to ensure precise and consistent power output measurements. The calibration followed the manufacturer’s standard procedures, focusing on verifying the drag factor and ensuring that the monitor’s internal system was functioning within specified tolerances. Specifically, the drag factor was adjusted based on each participant’s body weight and rowingte or build upon this study with confi technique, such as stroke length and power distribution. This process was completed using the built-in calibration function of the Concept2, which checks the accuracy of power measurements across different resistance levels, ensuring reliable data throughout the intervention period. Calibration was performed at the beginning of each training session to account for any potential drift in device readings over time.

In addition to power output monitoring, heart rate was continuously recorded using the Polar H10 telemetry system, providing accurate insights into the participant’s cardiovascular exertion. This dual monitoring approach allowed us to track both external load (power output) and internal load (heart rate), providing comprehensive verification of intensity.

Before the main intervention, a pilot session was conducted for all participants. During this preliminary session, participants performed multiple 1-min intervals while both heart rate and power output were monitored in real time. This pilot test helped confirm each participant’s ability to sustain maximal effort and ensured they met the prescribed intensity for each interval. Any discrepancies in effort were addressed by adjusting intensity targets on an individual basis, ensuring consistency across all participants.

This dual-system monitoring of real-time power output and heart rate, along with the pre-intervention pilot session, ensured that intensity was consistently maintained throughout each training session. The combined monitoring approach allowed for precise control of training load, ensuring that maximal intensity was sustained and minimizing individual variability, thereby optimizing the training stimulus.

### Rationale for anaerobic power reserve (APR) selection

The APR was calculated based on the difference between MAP and MSP, as previously outlined by Sandford et al. (2021). This calculation method provides a standardized measure of the available anaerobic power, which is crucial for assessing performance during high-intensity efforts such as sprints or maximal rowing intervals (Jamnick et al., 2020; Wang and Zhao, 2023). By using APR as the basis for training intensity, we ensured that the rowers’ anaerobic capacities were effectively challenged during the training sessions while maintaining an appropriate balance with their aerobic contributions. This approach has been shown to optimize the engagement of both energy systems, enhancing performance and physiological adaptations across different athletes.

## Results

**FIGURE 1:**
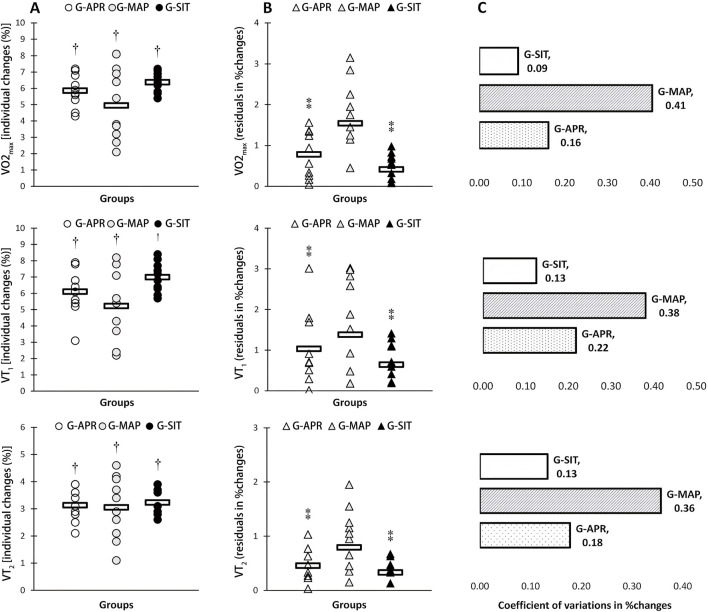
Individual changes **(A)** variability in adaptive responses individual changes **(B)** and coefficient of variations (CV) in mean group changes **(C)** in maximum oxygen uptake (V̇O2max), first ventilatory threshold (VT1), and second ventilatory threshold (VT2) in response to high-intensity interval interventions individualized using anaerobic power reserve (G-APR), maximal aerobic power (G-MAP), and maximal exertion (G-SIT). Circles indicate individual percent changes from baseline (X-axes), and horizontal bars represent the group mean response. † Denotes significant difference vs.pretraining (p ≤ 0.05). ⁑ Denotes significant difference vs G-MAP (p ≤ 0.05).

**FIGURE 2:**
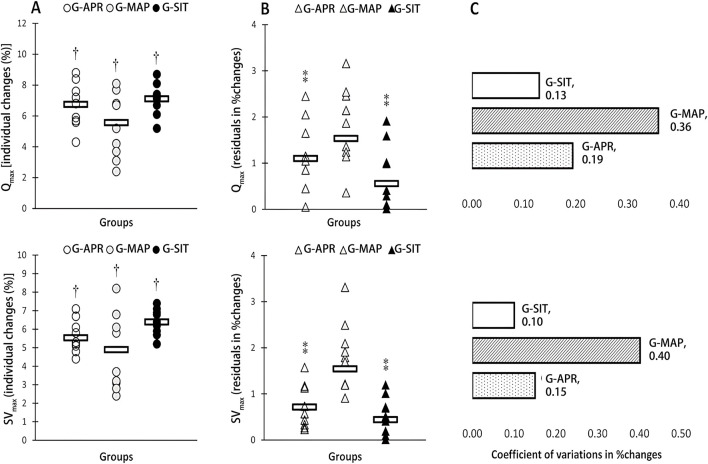
Individual changes **(A)** variability in adaptive responses in individual changes **(B)** and coefficient of variations (CV) in mean group changes **(C)** in maximal cardiac output (Q̇ max), and stroke volume (SVmax) in response to high-intensity interval interventions individualized using anaerobic power reserve (GAPR), maximal aerobic power (G-MAP), and maximal exertion (G-SIT). Circles indicate individual percent changes from baseline (X-axes), and horizontal bars represent the group mean response. † Denotes significant difference vs pre-training (p ≤ 0.05). ⁑ Denotes significant difference vs G-MAP (p ≤ 0.05).

**FIGURE 3:**
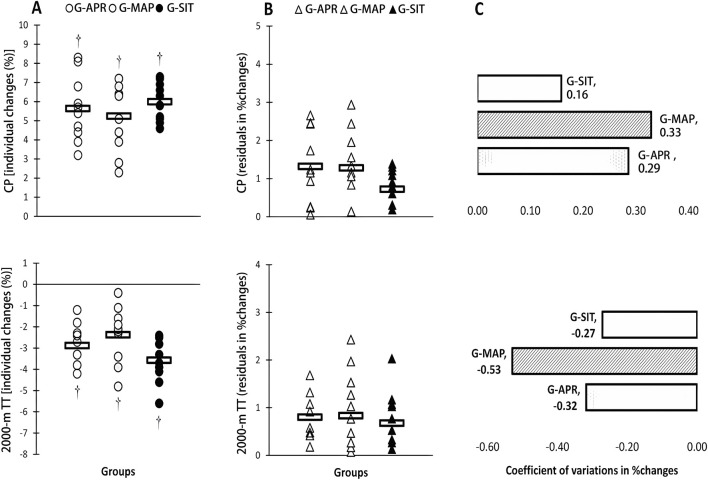
Individual changes **(A)** variability in adaptive responses in individual changes **(B)** and coefficient of variations (CV) in mean group changes **(C)** in critical power (CP), and 2,000-m time trial performance in response to high-intensity interval interventions individualized using anaerobic power reserve (GAPR), maximal aerobic power (G-MAP), and maximal exertion (G-SIT). Circles indicate individual percent changes from baseline (X-axes), and horizontal bars represent the group mean response. † Denotes significant difference vs pre-training (p ≤ 0.05)

**FIGURE 4:**
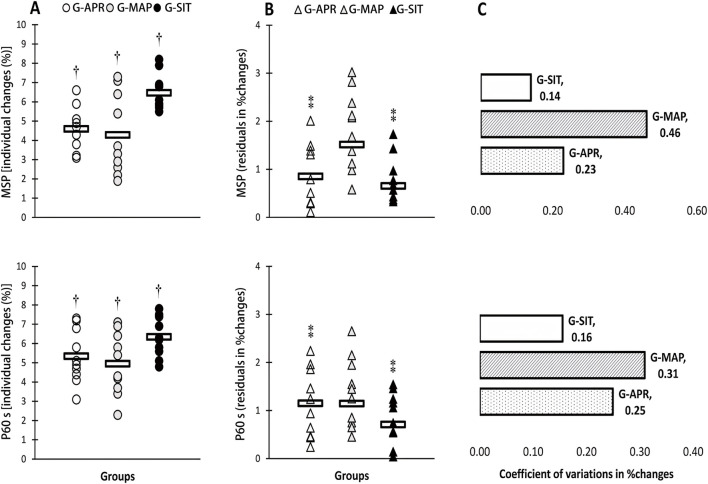
Individual changes **(A)** variability in adaptive responses in individual changes **(B)** and coefficient of variations (CV) in mean group changes **(C)** in maximal sprint power (MSP), and 60-s power (P60 s) in response to high-intensity interval interventions individualized using anaerobic power reserve (GAPR), maximal aerobic power (G-MAP), and maximal exertion (G-SIT). Circles indicate individual percent changes from baseline (X-axes), and horizontal bars represent the group mean response. † Denotes significant difference vs pre-training (p ≤ 0.05).

Table 2 summarizes the physiological changes observed in the three intervention groups: G-APR, G-MAP, and G-SIT. Significant improvements were observed across all groups for VO_2_max, ventilatory thresholds (VT1, VT2), Qmax, SVmax, MSP, P60s, CP, and 2,000-m TT performance (p < 0.05). As shown in Figure 1, the G-SIT group consistently demonstrated the largest relative gains, with VO_2_max increasing by 6.3% (from 51.9 ± 3.2 to 55.2 ± 3.3 mL·kg^−1^·min^−1^, effect size = 1.05, 95% CI [0.57, 1.53]). This improvement is greater than that observed in the G-APR and G-MAP groups, whose effect sizes were 0.89 and 0.75, respectively. As shown in Figure 2, Qmax increased significantly by 6.4% in the G-SIT group (effect size = 1.15, 95% CI [0.66, 1.64]), while SVmax showed a 6.5% increase (effect size = 0.79, 95% CI [0.32, 1.26]). These changes indicate improved cardiovascular efficiency, with enhanced oxygen delivery capabilities during high-intensity exercise—critical for maintaining power during rowing. In the 2,000-m TT, as shown in Figure 3, the G-SIT group exhibited the largest reduction of 3.7% in completion time (from 385.9 ± 6.2 s to 372.1 ± 5.6 s, p = 0.002, effect size = 0.86, 95% CI [0.39, 1.33]), emphasizing the protocol’s effectiveness in improving rowing efficiency. Improvements in anaerobic power metrics, including MSP (6.4%, effect size = 0.61, 95% CI [0.18, 1.04]), as shown in Figure 4, P60s (6.2%, effect size = 0.72, 95% CI [0.27, 1.17]), and CP (6.0%, effect size = 0.71, 95% CI [0.25, 1.18]), highlight the G-SIT protocol’s effectiveness in enhancing short-duration, high-intensity output, which is essential for race start and sprint phases.

**TABLE 2:** Changes in measured variables over the training period.

Group																		
	G-APR						G-MAP						G-SIT					
	Pre-test	Post-test	%Δ	*P*	Effect Size	95% CI	Pre-test	Post-test	%Δ	*P*	Effect Size	95% CI	Pre-test	Post-test	%Δ	*P*	Effect Size	95% CI
VO_2max_ (ml·kg^−1^·min−^1^)	51.9 ± 2.3	55.0 ± 2.4	5.9^†^	0.002	0.89	(0.42, 1.36)	52.5 ± 3.0	55.1 ± 3.1	4.9^†^	0.005	0.75	(0.30, 1.20)	51.9 ± 3.2	55.2 ± 3.3	6.3^†^	0.001	1.05	(0.57, 1.53)
VT_1_ (%VO2max)	73.6 ± 4.1	78.1 ± 4.9	6.1^†^	0.004	0.82	(0.35, 1.29)	72.5 ± 5.8	76.3 ± 5.9	5.2^†^	0.007	0.7	(0.24, 1.16)	74.6 ± 4.2	79.9 ± 4.4	7.1^†^	0.002	0.96	0.48,1.44)
VT_2_ (%VO2max)	88.5 ± 6.1	91.7 ± 6.8	3.6^†^	0.002	0.58	(0.15,1.01)	87.8 ± 5.4	90.5 ± 6.2	3.1^†^	0.005	0.5	(0.08,0.92)	87.4 ± 4.7	90.9 ± 5.0	4.0^†^	0.001	0.73	(0.28,1.18)
Q_max_ (L·min−^1^)	31.0 ± 1.6	33.1 ± 1.7	6.7^†^	0.001	1.24	(0.74,1.74)	31.5 ± 2.1	33.3 ± 2.4	5.7^†^	0.004	0.96	(0.48,1.44)	30.9 ± 2.1	33.2 ± 2.2	6.4^†^	0.001	1.15	(0.66,1.64)
SV_max_ (mL·beat^-1^)	158.2 ± 17.3	167.0 ± 18.2	5.5^†^	0.002	0.64	(0.20,1.08)	159.6 ± 17.5	167.4 ± 18.8	4.9^†^	0.008	0.56	(0.13,0.99)	160.2 ± 18.9	170.6 ± 19.9	6.5^†^	0.003	0.79	(0.32,1.26)
MSP(W)	737.9 ± 53.2	771.7 ± 56.2	4.6^†^	0.002	0.61	(0.18,1.04)	716.9 ± 61.6	747.6 ± 68.4	4.2^†^	0.005	0.55	(0.13,0.97)	723.1 ± 65.8	769.8 ± 70.4	6.4^†^	0.002	0.78	(0.30,1.26)
P60s(W)	653.7 ± 44.6	688.2 ± 42.4	5.2^†^	0.001	0.88	(0.41,1.35)	618.8 ± 59.4	649.3 ± 61.2	4.9^†^	0.002	0.72	(0.27,1.17)	627.8 ± 61.0	667.2 ± 58.6	6.2^†^	0.001	0.93	(0.46,1.40)
CP(W)	396.5 ± 31.1	418.7 ± 32.4	5.6^†^	0.002	0.85	(0.38,1.32)	386.0 ± 39.5	405.1 ± 38.3	4.9^†^	0.003	0.71	(0.25,1.17)	376.5 ± 28.4	399.1 ± 28.7	6.0^†^	0.001	0.97	(0.49,1.45)
2,000-m TT (sec)	389.4 ± 6.4	378.9 ± 6.0	−2.7^†^	0.001	0.63	(0.19,1.07)	389.3 ± 4.0	380.1 ± 7.7	−2.4^†^	0.006	0.52	(0.10,0.94)	385.9 ± 6.2	372.1 ± 5.6	−3.7^†^	0.002	0.86	(0.39,1.33)

Values are means ± SD.

VO2max, maximum oxygen uptake; VT_1_, first ventilatory threshold; VT_2_, second ventilatory threshold; Qmax, maximal cardiac output; SVmax, maximal stroke volume; MSP.

maximal sprinting power; P60s, 60-s power; CP, critical power; TT, time trial.

† Significantly different from baseline value (P < 0.05).

## Discussion

This study is the first to compare the consistency of physiological and performance adaptations from HIIT interventions individualized by APR, MAP, and SIT in elite male rowers. All three interventions led to significant improvements in cardiorespiratory fitness (VO_2_max, Q̇max, SVmax, VT1, VT2), anaerobic power (MSP, P60s, CP), and 2,000-m time trial performance. The most significant finding was that G-APR and G-SIT produced lower variability in individual changes and coefficients of variation (CVs) in group mean changes compared to G-MAP. Furthermore, G-SIT resulted in notably lower CVs for adaptive changes than G-APR.

The reduced variability observed with G-APR is consistent with prior studies, which have shown more uniform adaptations in cardiorespiratory fitness and anaerobic power following APR/ASR-based HIIT (Wang and Zhao, 2023; Luo et al., 2024; Du and Tao, 2023; Dai and Xie, 2023; Wang and Ye, 2024). This study also compares an APR-based HIIT with a volume-matched SIT protocol, demonstrating that SIT led to more consistent adaptations across participants. Lower CVs in group mean changes further support this conclusion. In nearly all measured variables, G-SIT exhibited smaller residual variability in adaptive changes than G-APR, supporting the idea that using fixed percentages of reference intensities often fails to standardize metabolic stress across participants (McPhee et al., 2010; Mann et al., 2013; Bassett and Howley, 2000; Thurlow et al., 2024), leading to greater variability in responses (Mann et al., 2014; Meyler et al., 2021; Meyler et al., 2023).

It is important to note that while both G-APR and G-SIT protocols utilized relative intensities, the key distinction between the two lies in the level of intensity applied. G-APR was set at 30% of the anaerobic power reserve, whereas G-SIT required maximal exertion, or “all-out” efforts. The reduced variability in adaptive responses observed in G-SIT is attributed not to the method of calculating intensity, but to the higher level of intensity applied during the interventions. By using SIT, which relies heavily on anaerobic metabolism, variability in adaptive responses decreases as SIT demands maximum exertion, thereby engaging participants’ full physiological capacity. Previous studies have shown that physiological variability decreases at higher exercise intensities, with the least variation occurring under maximal conditions (Bagger et al., 2023; O'Grady, 2021). Our findings confirm this, as G-SIT demonstrated reduced inter-individual variability across all measured variables compared to G-APR and G-MAP.

The reduced variability in MSP adaptations in G-APR and G-SIT compared to G-MAP further underscores the effectiveness of these protocols in minimizing differences in adaptive responses. Equal engagement of the anaerobic metabolic system across participants likely contributed to this consistency. However, G-SIT produced lower residual variability and CVs in MSP, P60s, and CP than G-APR, suggesting that APR-based interventions may not fully account for all factors influencing load (Cavar et al., 2019). Maximal exertion in SIT appears to maximize anaerobic system engagement, leading to more uniform improvements in anaerobic power. Despite these findings, no significant differences were observed in residual variability for P60s, CP, or 2,000-m time trial performance, suggesting that additional factors like motivation, endurance, and muscle composition may influence performance in these tests (Wang and Ye, 2024).

The G-SIT protocol demonstrated superior improvements in both aerobic and anaerobic power metrics, including VO_2_max, Q̇max, SVmax, MSP, and 2,000-m rowing performance, surpassing other protocols in enhancing elite rowers’ physiological adaptations. This effectiveness is attributed to maximal anaerobic effort, which enhances type II muscle fiber recruitment, neuromuscular efficiency, and phosphocreatine availability. Additionally, G-SIT showed lower variability in adaptive responses, suggesting greater consistency across individuals, which is advantageous in team sports (Hahn et al., 2000; Saltin and Calbet, 2006; Bray et al., 2009). The advantage of prescribing maximal intensity lies in its simplicity, as it reduces the need for complex intensity calibration and allows for a straightforward application across all participants. This approach ensures a high level of engagement from the anaerobic system, promoting effective physiological adaptations.

## Conclusion

This study examined the adaptive effects of three HIIT protocols on elite male rowers: APR, MAP, and SIT. All groups showed significant improvements in cardiorespiratory fitness, anaerobic power, and 2,000-m rowing performance, with SIT demonstrating the most consistent adaptations and lowest variability in key metrics, including VO_2_max, second ventilatory threshold, Qmax, SVmax, and sprint power. The enhanced consistency in the SIT group may be attributed to the psychological demands of maximal exertion, which likely minimized discrepancies in effort among participants. Differences in adaptive responses between APR and MAP suggest that individual variability in anaerobic capacity significantly influences HIIT outcomes (Rosenblat et al, 2021). These findings underscore the importance of considering both physiological markers and motivational factors to optimize individualized HIIT protocols.

The practical significance of SIT was highlighted by its consistent improvements in both aerobic and anaerobic performance. Compared to APR and MAP, SIT led to reduced variability in adaptive responses, which is crucial for team sports where uniformity in performance is essential. The high exertion required in SIT likely optimized adaptations by engaging type II muscle fibers and enhancing cardiovascular efficiency, contributing to more reliable training outcomes across individuals. The uniformity achieved through SIT makes it a promising option for training protocols aimed at achieving similar levels of improvement among athletes.

## Limitations

This study faced several limitations, primarily in controlling exercise intensity during HIIT sessions. Despite monitoring heart rate and power output, maintaining consistent intensity across participants was challenging, particularly in SIT, where fatigue and motivation could vary significantly. Advanced monitoring techniques, such as real-time feedback and stricter adherence protocols, could help reduce these inconsistencies. Additionally, genetic variability complicated the interpretation of individual responses, particularly in VO_2_max and anaerobic power, though this should be considered an inherent factor rather than a specific limitation (Lippi et al., 2009; Bouchard et al., 1999). Furthermore, the relatively short intervention period may not fully capture long-term adaptations or potential plateaus.

## Future research

Future research should focus on the long-term applicability and sustainability of SIT and other HIIT protocols, consider genetic screening for personalized training plans, and explore hybrid training methods to enhance athlete performance. It would also be valuable to include a broader range of physiological indicators for a more comprehensive understanding of training adaptations. Additionally, studies should account for the impact of psychological factors on training responses, optimize the integration of HIIT across different training phases, extend research to athletes of varying proficiency levels, and utilize advanced technologies to improve the precision of training monitoring. These directions will deepen the understanding of HIIT effects and provide a scientific basis for sports training.

## Data Availability

The raw data supporting the conclusions of this article will be made available by the authors, without undue reservation.
