# Correction: Supramaximal interval training using anaerobic speed reserve or sprint interval training in rowers

**DOI:** 10.3389/fphys.2025.1671860

**Published:** 2025-08-20

**Authors:** Yu Tongwu, Zhong Jinghui, Ding Chuanwei, Zhang Zijian, Xu Yuxiong

**Affiliations:** ^1^ Anhui Communications Vocational and Technical College, Hefei, China; ^2^ Capital University of Physical Education and Sports, Beijing, China

**Keywords:** high-intensity interval training, sprint interval training, aerobic fitness, anaerobic power, performance variability, rowing

In the published article, there was an error in **affiliation(s)** [Yu Tongwu1,2]. Instead of “Yu Tongwu1,2”, it should be “Yu Tongwu1”.

The original article has been updated.

